# Social Engagement and Its Change are Associated with Dementia Risk among Chinese Older Adults: A Longitudinal Study

**DOI:** 10.1038/s41598-017-17879-w

**Published:** 2018-01-24

**Authors:** Zi Zhou, Ping Wang, Ya Fang

**Affiliations:** 1 State Key Laboratory of Molecular Vaccinology and Molecular Diagnostics, School of Public Health, Xiamen University, Xiamen, Fujian 361102 China; 2 Key Laboratory of Health Technology Assessment of Fujian Province University, School of Public Health, Xiamen University, Xiamen, Fujian 361102 China

## Abstract

This study aimed to examine the association of social engagement (SE) and changes in SE with the risk of dementia among older adults in China. Data were collected from the 2002, 2005, 2008/2009, and 2011/2012 waves of the Chinese Longitudinal Healthy Longevity Survey (CLHLS). Random-effects logistic regression models were used to examine the association of SE and changes in SE with the risk of dementia. Of the 7511 older Chinese adults aged 65 years and over, 338 developed dementia during the 9-year follow-up. SE was associated with dementia risk after adjusting for sociodemographic characteristics, lifestyles and health status (odds ratio (*OR*) = 0.71, 95% confidence interval (*CI*) = 0.63–0.81). Participants with consistently high or increased SE had a lower risk of dementia than those with consistently low SE ((*OR* = 0.14, 95% *CI* = 0.06–0.28 and *OR* = 0.33, 95% *CI* = 0.23–0.48, respectively). Higher SE can reduce the risk of dementia. Furthermore, consistently high or increasing SE is associated with a lower risk of dementia.

## Introduction

Dementia is an irreversible progressive disease that has no effective treatment^1^. A recent study has shown that 35.6 million people worldwide suffer from dementia, and this number is predicted to double by 2030 and more than triple by 2050^2^. China had the largest number of persons with dementia in 2010 with 9.19 million^3^. Additionally, the increasing prevalence of dementia may lead to a sharp rise in the social and economic burdens of the disease. The total worldwide societal cost of dementia was estimated to be $315 billion in 2005 and $422 billion in 2009, indicating an increase of 34% between 2005 and 2009. The societal costs of dementia in China were $41 billion, leading China to become the country with the third-highest societal costs of dementia following the United States and Japan. China, as the largest developing country, will face grave challenges in preventing and controlling dementia.

Accordingly, the risk factors of dementia must be considered. Several factors, including diabetes^4^, disability in activities of daily living (ADL)^5^ and exercise^6^, have been associated with the risk of dementia. Notably, fewer studies have examined the association between social engagement (SE) and the risk for the onset of dementia, and the findings have been inconsistent. SE refers to the maintenance of social connections and participation in social activities^7^. A few prospective studies of older adults have indicated that the risks of cognitive decline and dementia are increased in individuals who are isolated, lack physical activity and have poor social networks and support^8–15^. A longitudinal study from the Kungsholmen Project^16^ showed that engagement in social and leisure activities in late life might decrease the risk of dementia in older adults because participation in productive or social activities could potentially sustain individuals’ senses of self-efficacy and thus improve the health statuses of older adults. Additionally, engaging in intellectually challenging activities may promote or enhance cognitive performance. The Envejecer en Leganes study^17^ showed that good social relationships with friends and relatives might generate continued mental stimulation and better cognitive strategies in late life. Moreover, these relationships may increase synaptic density and neural growth, which can delay cognitive decline or prevent pathological processes^18,19^. Similar results were obtained from Dutch^8^ and Swedish^10^ studies on the elderly. However, these findings are in contrast with the results of previous studies^20,21^ showing that social relationships are not associated with an decreased risk of dementia or Alzheimer disease (AD) in the elderly.

In addition to these controversies, most epidemiological studies documenting the association between SE and incident dementia have examined only one baseline measurement of SE and related this measure to the subsequent dementia risk. Conversely, these studies have rarely investigated changes in SE after the baseline. This type of investigation is important because many people change their SE level throughout life, and these changes may affect the risk of dementia. To the best of our knowledge, only one study has evaluated the association between changes in SE from midlife to late life and the risk of dementia with a focus on old men^22^. Saczynski *et al*.^22^ indicated that only decreasing SE from midlife to late life was associated with an increased risk of dementia, whereas increasing SE was not associated with a reduced risk of dementia among Japanese-American men.

The objective of this study is to examine the association of SE and changes in SE with the risk of dementia in a cohort during a 9-year follow-up. The sample was derived from the Chinese Longitudinal Healthy Longevity Survey (CLHLS).

## Methods

### Data and study population

The data in this study came from a large nationally representative survey (the CLHLS), which included waves in 1998, 2000, 2002, 2005, 2008/2009, and 2011/2012. The sample was randomly stratified and selected from 22 of 31 provinces, which represented an extensive 85% of the total Chinese population. The first two waves (1998 and 2000 waves) of the CLHLS were removed because they mainly targeted the population aged 80 years and over. Therefore, the sample aged 65 years and over included in the 2002 wave was selected as the baseline in this study. Three follow-ups (the 2005, 2008/2009, and 2011/2012 waves) were completed in the 9-year cohort study. Duke University Health System’s Institutional Review Board (IRB) reviewed and provided ethical approval of this study. Detailed information about the CLHLS study design and data quality is reported elsewhere^23^.

**Figure 1:**
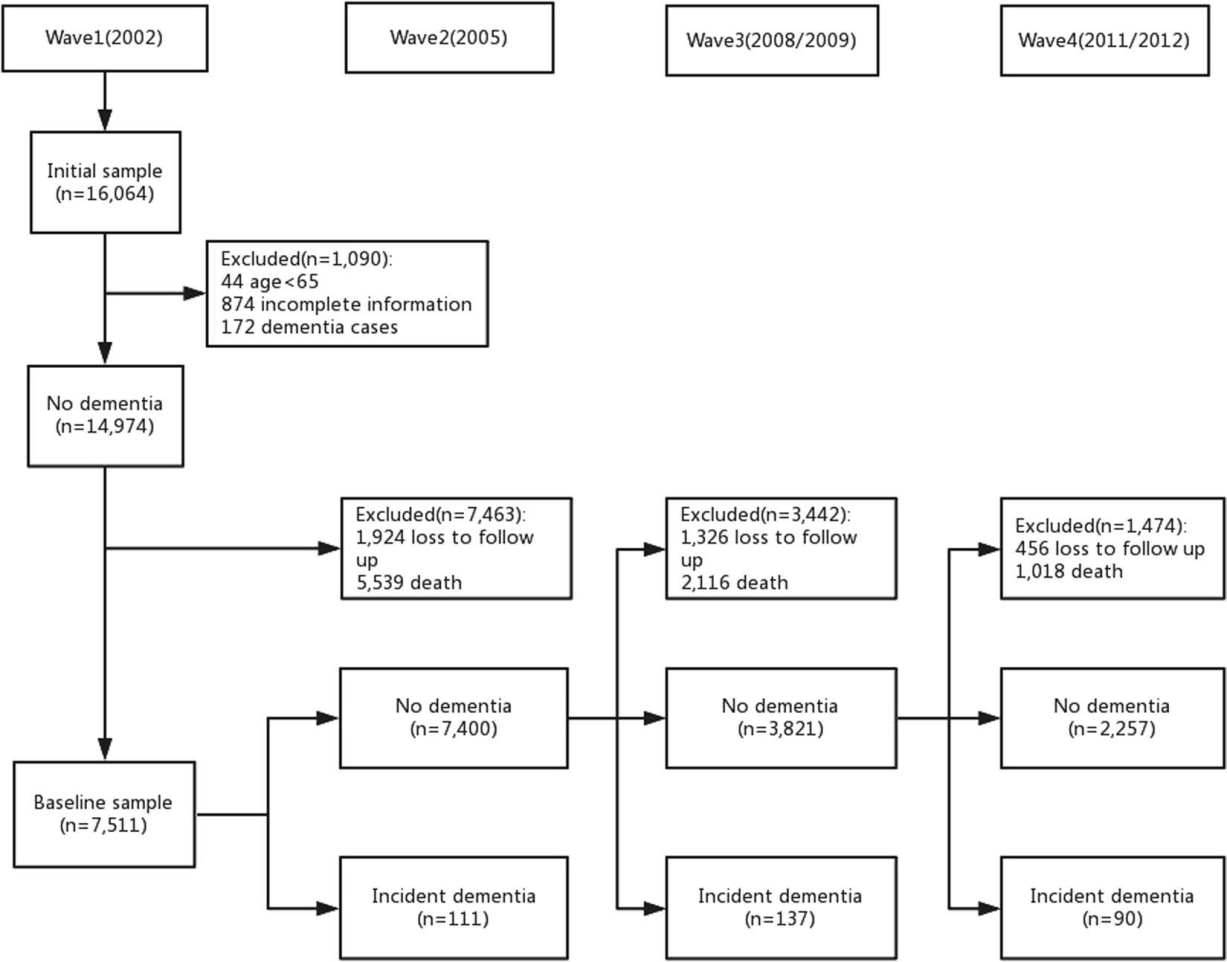
Flow chart of sample selection.

The subjects of this study were limited to those who were aged 65 years and over and did not have dementia at baseline. To analyze the risk of dementia, the subjects had to have received at least one follow-up visit. For the SE analyses, we limited the subjects to those who had valid information and included data up until the time that they developed dementia. Detailed information about the sample selection process is provided in Fig. 1.

### Measures

#### Dementia

The diagnosis of dementia was determined according to two items (1. Are you suffering from dementia? and 2. Was it diagnosed by a hospital?). We considered the participants to be suffering from dementia only when they or their caregiver responded “yes” to both items (0 = no dementia, 1 = suffering from dementia). Those who suffered from dementia before death were diagnosed in the same manner.

#### Social engagement

According to a previous study^24^, information on the quantity of SE consists of five dichotomous variables: 1) marital status (0 = not/no longer married, 1 = married); 2) living arrangement (0 = living alone, 1 = living with family, friends or other persons); 3) the availability of help when required (0 = no, 1 = yes), according to the item “Do you have someone you can ask for help when you have problems/difficulties?”; 4) the availability of a confidant, in response to the question “Do you have someone to talk to when you need to share some of your thoughts?”, which was coded as no (0) or yes (1); and 5) participation in social activities, which was categorized as no (0) or yes (1). Finally, the SE score was calculated as the sum of the five variables (from 0 to 5, with a higher score indicating greater SE).

#### Change in SE during two successive waves

A new variable was developed by combining the participants’ responses. SE was divided into three categories: a low level of SE (scores of 0–2), a medium level of SE (scores of 3) and a high level of SE (scores of 4–5). SE changes were classified into five categories according to the change in SE across two successive waves: 1) consistently low SE; 2) decreasing SE; 3) increasing SE; 4) consistently medium SE; and 5) consistently high SE.

#### Other covariates

According to previous studies, potentially related factors were classified into three groups as follows: sociodemographic characteristics, lifestyles, and health status.

The sociodemographic characteristics included age, literacy, type of residence, and engagement in physical labor. Age was classified into three categories (65 to 74, 75 to 84, and ≥85 years). The respondents were classified as illiterate (0) or literate (1) based on whether they had received an education. Gender was categorized in male (0) and female (1). The type of residence was defined as urban (0) or rural (1). Engagement in physical labor was categorized as no (0) or yes (1).

For lifestyles, smoking, drinking and exercise were categorized into no (0) and yes (1) responses according to their present statuses.

For the health status, cognitive functioning was assessed using the Mini Mental State Examination^25^. The total score of the scale is 30, with lower scores indicating poorer cognitive functioning. An adapted version of the scale was used to measure ADL disability, and the scale contained the six daily tasks of dressing, eating, toileting, bathing, engaging in indoor activities, and continence^26^. We scored participants as 0 when they responded with complete self-help to all questions of the scale; otherwise, they were scored as 1. Information on hypertension, diabetes and stroke/cerebrovascular disease was determined similarly to the information on dementia (0 = no, 1 = yes).

### Data analyses

Descriptive statistics were utilized to present the sample characteristics, and the Chi-square test was performed for comparisons between participants who developed dementia and those who did not.

Random-effects logistic regression models were employed to examine the association of SE and changes in SE with the risk of dementia and to estimate the odds ratio (OR) and 95% confidence interval (CI). To ensure that the exposure preceded the outcome, dementia onset was modeled on the SE exposure from one wave before the dementia measurement. For example, for the dementia outcomes reported in the 2005 wave, 2002 was considered the SE exposure wave. For the dementia outcomes reported in the 2008/2009 wave, 2005 was the exposure wave, and so forth. Hence, three periods (2002–2005, 2005–2008/2009, and 2008/2009–2011/2012) were analyzed in this study.

Three models were developed in this study. Sociodemographic characteristics (age, literacy, gender, type of residence, and engagement in physical labor) were first entered into the model 1, lifestyle factors (smoking, drinking, and exercise) were added in model 2, and the final model (model 3) adjusted for the variables in model 2 plus the health status (cognitive functioning, ADL disability, hypertension, diabetes, and stroke/cerebrovascular disease). Missing data (for the imputed 9 variables: median missing percentage 1.09%, minimum 0.09%, and maximum 4.65%) were imputed using the NORM program^27^.

Sensitivity analyses were performed to examine whether potential bias could have been introduced by individuals who were lost to follow-up or died. For the censored samples, we assumed that people who had poor cognitive functioning (Mini Mental State Examination scores less than 1.5 standard deviations below the mean age-education-adjusted scores at their last wave) would develop dementia in the next wave based on previous studies^28,29^. The analyses were repeated to assess whether the association between SE and dementia risk changed. All analyses were performed using Stata version 13.0 (StataCorp; College Station, TX, USA).

## Results

### Subject characteristics

The 7511 participants contributed 13,816 observations, and 338 cases developed dementia over the 9-year follow-up. The participants who developed dementia were older, more likely to be female and not engaged in physical labor, resided in urban, and spent less time exercising; additionally, they were more likely to suffer from poor cognitive functioning, ADL disability, hypertension, diabetes, and stroke/cerebrovascular disease (*p* < 0.05). Moreover, the subjects who developed dementia had a lower level of SE than those who did not (*p* < 0.001), and SE measurements during two successive waves were more likely to decrease or remain low (*p* < 0.001) (Table 1).

**Table 1:** Participant Characteristics by Dementia Status, China, 2002–2011 (*n* = 13,816).

**Variables**	**Non-dementia (** ***n*** ** = 13,478)** ***n*** **(%)**	**Dementia (** ***n*** ** = 338)** ***n*** **(%)**	***P***
SE			<0.001
1	867(6.65)	32(10.32)	
2	2,474(18.98)	103(33.23)	
3	4,829(37.05)	123(39.68)	
4	3,584(27.50)	43(13.87)	
5	1,281(9.83)	9(2.90)	
Age (years)			<0.001
65~	5,382(39.93)	66(19.53)	
75~	4,177(30.99)	86(25.44)	
85~	3,919(29.08)	186(55.03)	
Female	7,258(53.85)	202(59.76)	0.031
Rural	7,759(57.57)	170(50.30)	0.008
Literate	5,945(44.11)	141(41.72)	0.381
Physical labor	11,110(82.43)	255(75.44)	0.001
Smoking	2,926(21.71)	62(18.34)	0.138
Drinking	3,003(22.28)	65(19.23)	0.183
Exercise	5,199(38.57)	80(23.67)	<0.001
Hypertension	2,252(16.71)	72(21.30)	0.026
Diabetes	294(2.18)	14(4.14)	0.016
Stroke/Cerebrovascular disease	612(4.54)	41(12.13)	<0.001
Cognitive functioning, mean (SD)	24.74(6.70)	17.72(10.22)	<0.001
ADL disability	1,739(12.90)	124(36.69)	<0.001
Changes in SE			<0.001
Consistently high SE	1,521(11.29)	8(2.37)	
Consistently medium SE	1,196(8.87)	7(2.07)	
Increasing SE	2,427(18.01)	47(13.91)	
Decreasing SE	6,783(50.33)	172(50.89)	
Consistently low SE	1,551(11.51)	104(30.77)	

Note: Abbreviations: ADL, activities of daily living; SE, social engagement.

### The association between SE and dementia risk

Table 2 presents the relationship between SE and dementia risk during the 9-year follow-up. The analysis showed that the fit of the model was significantly improved in models 2 and 3. The random-effects logistic regressions showed that SE was significantly associated with dementia risk in the three models.

**Table 2:** The Association between Social Engagement and Dementia Risk, China, 2002–2011.

**Variables**	**Model 1**		**Model 2**		**Model 3**	
	*OR*	95% *CI*	*OR*	95% *CI*	*OR*	95% *CI*
SE	0.63^**^	0.56, 0.71	0.65^**^	0.58, 0.73	0.71^**^	0.63, 0.81
Age (years)	2.00^**^	1.72, 2.32	1.96^**^	1.68, 2.28	1.50^**^	1.26, 1.78
Female	0.97	0.74, 1.26	0.96	0.72, 1.27	0.90	0.68, 1.20
Rural	0.74^*^	0.58, 0.94	0.67^**^	0.53, 0.85	0.70^**^	0.55, 0.90
Literate	1.20	0.91, 1.58	1.27	0.96, 1.67	1.45^**^	1.09, 1.93
Physical labor	0.67^**^	0.51, 0.88	0.65^**^	0.49, 0.85	0.72^*^	0.54, 0.95
Smoking			1.14	0.83, 1.56	1.17	0.85, 1.62
Drinking			1.04	0.77, 1.41	1.18	0.86, 1.60
Exercise			0.54^**^	0.41, 0.71	0.66^**^	0.50, 0.87
Hypertension					1.26	0.94, 1.71
Diabetes					1.99^*^	1.10, 3.60
Stroke/Cerebrovascular disease					2.29^**^	1.55, 3.39
Cognitive functioning					0.94^**^	0.93, 0.95
ADL disability					1.62^**^	1.23, 2.13

Note: *P < 0.05; **P < 0.01; ***P < 0.001. All *P* values are 2 sided.

Abbreviations: OR, odds ratio; CI, confidence interval; SE, social engagement; ADL, activities of daily living.

When sociodemographic characteristics were controlled in model 1, the association of SE with the risk of dementia was significant (*OR* = 0.63, 95% *CI* = 0.56–0.71). The association remained robust after adjusting for lifestyle factors in model 2 (*OR* = 0.65, 95% *CI* = 0.58–0.73). Persons with a 1-unit increase in their SE score had an approximately 29% reduced risk of developing dementia during the 9-year follow-up (*OR* = 0.71, 95% *CI* = 0.63–0.81). Additionally, participants who were younger, literate, had higher cognitive functioning, resided in rural areas, engaged in physical labor, and exercised had a lower risk of dementia, whereas those who had an ADL disability, diabetes and stroke/cerebrovascular disease were more prone to develop dementia (Table 2).

### The association between changes in SE and dementia risk

The associations between changes in SE and dementia risk are shown in Table 3. People whose SE remained high or medium had a significantly lower risk of dementia than those whose SE remained low (*OR* = 0.14, 95% *CI* = 0.06–0.28 and *OR* = 0.12, 95% *CI* = 0.06–0.26, respectively) or decreased (*OR* = 0.29, 95% *CI* = 0.14–0.60 and *OR* = 0.26, 95% *CI* = 0.12–0.56, respectively). Increasing SE was associated with lower risk of dementia than consistently low SE (*OR* = 0.33, 95% *CI* = 0.23–0.48). However, consistently high SE across two successive interviews did not lead to a lower risk of dementia compared to consistently medium SE (*OR* = 1.12, 95% *CI* = 0.40–3.12).

**Table 3:** The Association between Changes in Social Engagement and the Dementia Risk, China, 2002–2011.

References	Changes in SE							
	Consistently high SE		Consistently medium SE		Increasing SE		Decreasing SE	
	*OR*	95% *CI*	*OR*	95% *CI*	*OR*	95% *CI*	*OR*	95% *CI*
Consistently medium SE	1.12	0.40, 3.12						
Increasing SE	0.41^*^	0.19, 0.87	0.36^*^	0.16, 0.81				
Decreasing SE	0.29^***^	0.14, 0.60	0.26^***^	0.12, 0.56	0.72	0.51, 1.00		
Consistently low SE	0.14^***^	0.06, 0.28	0.12^***^	0.06, 0.26	0.33^***^	0.23, 0.48	0.47^***^	0.36, 0.60

Note: Adjusted for Age (years), Female, Rural, Literate, Physical labor, Smoking, Drinking, Exercise, Hypertension, Diabetes, Stroke/Cerebrovascular disease, Cognitive functioning, and ADL disability. (See Table 1 for definitions).

*P < 0.05; **P < 0.01; ***P < 0.001. All *P* values are 2 sided.

Abbreviations: OR, odds ratio; CI, confidence interval; SE, social engagement.

The sensitivity analysis showed that higher SE had a robust association with dementia risk (*OR* = 0.94, 95% *CI* = 0.90–0.98). Additionally, no significant changes were found in the association between changes in SE and the risk of dementia (data not shown).

## Discussion

This longitudinal study examined the association of SE and changes in SE with dementia risk using a large population sample representative of Chinese older adults during a 9-year follow-up. After adjusting for potential confounders, such as sociodemographic characteristics, lifestyles, and health status, our results suggested that high SE could prevent or delay the incidence of dementia. Moreover, sustaining a consistently high SE or increasing SE was associated with a lower risk of dementia.

The findings of the association between SE and the risk of dementia were consistent with several prospective studies on older adults that indicated the predictive value of social relationships for the onset of dementia^8–11,14,16,22^. We found that the OR of dementia was 0.71 for those with higher SE, which was broadly consistent with the pooled relative risks of dementia in recent meta-analyses^9,11^. The majority of previous studies showed that low social participation was a risk factor for dementia. The results of the study by Wang *et al*. (2002) using Cox proportional hazards models showed that late life SE was associated with a decreasing risk of dementia after adjusting for potential confounders in a 6.4-year follow-up, albeit in a small sample^16^. A study of 2513 Japanese-American men by Saczynski *et al*.^22^ examined the relationship between SE and the incidence of dementia both in midlife and late life and showed that SE was associated with the incidence of dementia in older adults in late life but not in midlife^22^. The findings of the present study fall between those of other studies, with estimates of a higher risk of dementia following a lower level of social engagement. To the best of our knowledge, this study is the first to analyze the association between SE and dementia risk in an entire national population. Earlier studies used smaller populations, and thus, their results might be vulnerable to selection bias. Notably, random-effects logistic models were applied to fully utilize the time-dependent variables measured at each wave, which enabled us to obtain more accurate results during the 9 years of follow-up as opposed to only one baseline measurement of SE and to relate this finding to the subsequent risk of dementia.

The relationship between changes in SE and the risk of dementia has rarely been analyzed. To the best of our knowledge, only one study has explored the association of dementia risk with changes in SE from midlife to late life. The results of this study showed that men with decreased SE had a higher risk of dementia than those with consistently high SE; however, no significant differences were found between those with consistently low SE and consistently high SE^22^, which was inconsistent with our findings. Saczynski *et al*.^22^ targeted changes in SE with a long interval from midlife to late life but had no information on the period between the two time points; additionally, the authors used different measures for midlife to late life changes in social engagement. Therefore, the findings had low comparability with our findings. The present study analyzed the association of changes in SE with dementia risk, and the findings are beneficial for proposing effective policies to reduce the risk of dementia. Moreover, we could distinguish paths of changes in SE that might reflect health-related or other factors; for example, SE that remains low may reflect a certain personality type, which may be associated with dementia risk or an inability to relieve stress or loneliness^22^. Additionally, the association estimate for increasing SE more closely suggested that an intervention focused on enhancing SE would reduce the risk of dementia.

The association between SE and dementia risk may be explained by the following mechanisms. First, engagement in social activities may reduce the risk of dementia due to mental and intellectual stimulation^16^. Involvement in mental and intellectual activities may accelerate or preserve the brain reserve, especially in late life^16,18,30^. Second, a high level of SE may improve immune system functions^14^, which may delay the progression of dementia by affecting the cortical and limbic structure functions^31^. Third, a high level of SE could delay cognitive decline through effects on the cognitive status and positive emotional factors, such as social competence and self-esteem^17^.

This study had several strengths. First, this study included a national sample that represented 85% of older adults aged 65 years and over in China^23^, and this representativeness enhanced the significance of our findings for public health. Additionally, the 9-year follow-up of this cohort refutes to a certain extent the finding of occasional changes but a lifelong cumulative exposure of the association. Second, the random-effects logistic regression model capitalized on the use of time-varying variables in each wave to obtain more rational and accurate results over the 9 years of follow-up, which might offset the residual confounding associated with the use of only baseline variables, as shown in previous research. Finally, the association of dementia risk with changes in SE has rarely been examined in previous studies, especially in China. Designing effective interventions that aim to decrease the incidence of dementia would be beneficial.

Interpretation of our results should be considered within the context of a number of limitations of the study. First, the lack of a universal and available scale of SE in the CLHLS may limit our findings compared with previous studies. Nevertheless, because a standard scale has not necessarily been applied in older Chinese adults, we generated the SE variable after referring to an analogous study^24^. Second, considering the limited availability of data, the degree of satisfaction with SE could not be taken into account. A previous study indicated that the degree of satisfaction with SE was a more important factor than the number of social contacts^14,32^. Third, incident dementia is based on self- or proxy-reporting, which may have led to some bias. However, to a certain extent, the bias may be alleviated by the second question (“Have you been diagnosed with dementia by a physician?”). Moreover, the prevalence of self-reported dementia in this study is parallel to the prevalence in China^2^ and countries in East Asia^33^. Fourth, due to the lack of clinical classifications of dementia in the CLHLS, we were unable to account for the subclasses of dementia, such as Alzheimer’s disease or vascular dementia, which may have resulted in potential confounding bias in the strength of the association^20^. Fifth, the paucity of the dementia cases may have reduced the statistical power to test the association between SE and incident dementia. However, given the large sample size of the present study, this may not be a major problem. Finally, the possibility of reverse causation deserves attention because previous studies have shown that manifestations of a decrease in size of social networks related to deterioration in cognitive and physical functioning with insidious onset can occur years before a definitive diagnosis of dementia is made^34–36^. However, we excluded persons with incident dementia at baseline and controlled for baseline cognition functioning in all analyses. Thus, the prospective study design may have reduced the potential effects resulting from reverse causation.

In conclusion, low SE was associated with a higher risk of dementia even after adjusting for potential confounders. Persistently high SE could reduce the risk of dementia. Additionally, effective interventions to maintain a consistently high or increasing SE may be needed to reduce the risk of dementia. Future research should consider the degree of satisfaction with SE and the number of social contacts to examine the concrete mechanisms underlying the relationship between SE and dementia risk.
